# The TRANSNephro-study examining a new transition model for post-kidney transplant adolescents and an analysis of the present health care: study protocol for a randomized controlled trial

**DOI:** 10.1186/1745-6215-15-505

**Published:** 2014-12-23

**Authors:** Martin Kreuzer, Jenny Prüfe, Dirk Bethe, Charlotte Vogel, Anika Großhennig, Armin Koch, Martina Oldhafer, Marie-Luise Dierks, Urs-Vito Albrecht, Silvia Müther, Reinhard Brunkhorst, Lars Pape, Study group of the German Society for Pediatric Nephrology (Gesellschaft für Pädiatrische Nephrologie, GPN)

**Affiliations:** Department of Pediatric Kidney, Liver and Metabolic Diseases, Hannover Medical School, Carl-Neuberg-Strasse 1, 30625 Hannover, Germany; Institute of Biostatistics, Hannover Medical School, Carl-Neuberg-Strasse 1, 30625 Hannover, Germany; German Society of Transition Medicine, Carl-Neuberg-Strasse 1, 30625 Hannover, Germany; Department of Epidemiology, Social Medicine and Health System Research, Hannover Medical School, Carl-Neuberg-Strasse 1, 30625 Hannover, Germany; Berliner TransitionsProgramm (BTP), DRK-Kliniken Berlin Westend, Spandauer Damm 130, 14050 Berlin, Germany; KfH Center of Nephrology, Klinikum (Hospital) Region of Hannover, Podbielksistraße 380, 30625 Hannover, Germany; Division of Pediatric Nephrology, Center for Child and Adolescent Medicine, Heidelberg University Hospital, Im Neuenheimer Feld 672, 69120 Heidelberg, Germany; Peter L Reichertz Institute for Medical Informatics, Hannover Medical School, Carl-Neuberg-Strasse 1, 30625 Hannover, Germany

**Keywords:** Kidney transplantation, Transition, Immunosuppression, Telemedicine, Case management

## Abstract

**Background:**

The transition from pediatric to nephrology care is not yet a standardized procedure. The result is an increased risk of deteriorating transplant function, with the potential for premature transplant failure.

**Methods/Design:**

In phase I of this study, we shall evaluate the current patient transition situation in all existing German pediatric and nephrology departments (n = 17), including an evaluation of the views of physicians, nurses, and psychosocial support staff regarding transition. Phase II will be a prospective, randomized study in which we compare current unstructured transition (control group) to structured transition (intervention group). The structured transition approach integrates the core elements of the Berliner TransitionsProgramm in combination with two facilitating smartphone apps. The primary endpoint of this study will be therapy adherence, as reflected by group variation coefficients of immunosuppressive agent levels. As a secondary outcome, we will compare patients’ self-reported quality of life, satisfaction of patients and their parents with each transition model, and how patient-centered healthcare components are utilized. These secondary parameters will be assessed with established instruments or with instruments developed (and pilot tested) in phase I of the project.

**Discussion:**

The long-term goal of this work is to provide a model of structured transition from pediatric to adult care for adolescent nephrology patients, in order to improve transplant survival and patient wellbeing.

**Trial registration:**

Identifier: Clinicaltrials.gov: ISRCTN22988897, registered on 24 April 2014).

## Background

The transition of chronically ill adolescents from pediatric to adult medicine should conserve or improve their state of health. Such a transition can be considered successful if it promotes the patients’ health competence, supports their psychosocial rehabilitation, and improves their self-determination efficacy, including their ability to make decisions and communicate about their care. The overarching goal of transition is to enable patients to be as independent as possible and have the best possible quality of life. Achieving these goals requires, in addition to addressing medical issues, a multidimensional, multidisciplinary approach, including critical interventions in psychosocial, school-related, and occupational spheres [1].

Historically, a lack of cooperation among the various professionals involved in treatment and concomitant care has led to medical care issues for patients, which is particularly problematic in the transition phase. From an analysis of such deficiencies, it is possible to derive strategies for establishing a satisfactory patient-centered transition [1–4].

In nephrology in particular, the transition constitutes an important intersection in patient care [5, 6]. More transplanted kidneys are lost in patients aged between 16 and 21 years than in any other age group. Such graft failure requires a return to compulsory dialysis, which reduces quality of life and leads to considerable additional healthcare expense. Moreover, early graft losses increase mortality and shorten life expectancy. Some single-center transition models with transitional or adolescence-centered consulting hours (such as the Nephrology adolescent outpatient clinic of the Kuratorium for Dialysis and Transplantation in Hannover) have been established. They represent a new structural model for medical care in which there is a focus on prevention of non-adherence to reduce deterioration of kidney graft failure and, therefore, kidney diseases in this vulnerable age group [7, 8]. Training programs, such as ‘*Finally Grown Up*’ (‘*Endlich-Erwachsen*’) [9] and computer-based training courses [10], have been shown to produce some improvements in the transition of post-kidney transplant adolescents into adult medical care. For instance, the positive effects of ‘patient empowerment’ have been reported for adolescents receiving hyperphosphatemia therapy [11]. At present, it is not clear which, if any, transition models are applied in German pediatric nephrology centers.

**Figure 1:**
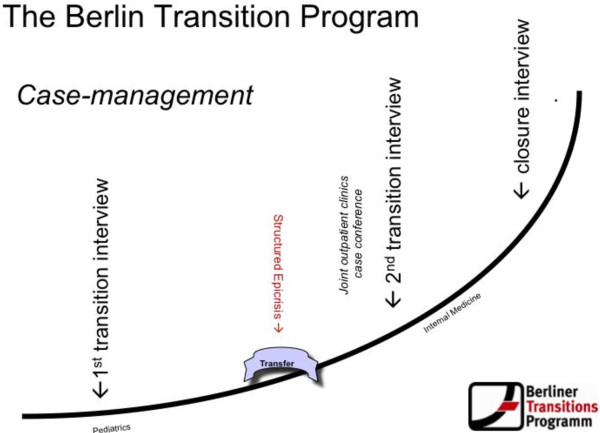
**Structure of the Berliner transitions programm.** eCRF, electronic case form.

The Berliner TransitionsProgramm is the first structured transition program in Germany to be financed by statutory health insurance (Figure 1). It regulates the transition of adolescents with different indications (currently, diabetes mellitus type 1, epilepsy, renal diseases, juvenile rheumatoid arthritis, inflammatory bowel diseases, and neuromuscular diseases) from pediatric to adult medicine over a period of two years. The program outlines the ways in which the course of this transition should proceed. Above all, it contains specific transitional measures for in-depth communication between the pre- and post-transition treating physicians, as well as conversations with adolescent patients and their parents, structured in terms of time and content. A case management protocol is used to support and ensure the unhindered flow of information and application of treatment measures [12].

The introduction of smartphone apps, which can be used to help monitor adolescent patients, may improve their adherence [13]. The rationale for using such apps is to interact with the patients through media that are familiar to them, enabling them to manage their disease even under the challenging conditions of adolescence. A willing acceptance of such high-tech instruments by adolescent patients can support and develop their competency in dealing with the disease.

We hypothesize that a structured transition, namely the Berliner TransitionsProgramm, complemented by telemedicine monitoring via smartphone apps, can facilitate the transition to adult care and improve the therapeutic adherence of adolescent patients living with a transplanted kidney. If correct, such an approach would improve post-transplant functioning and survival, and ultimately improve patient survival and wellbeing.

In this study, therefore, our first aim is to examine how many pediatric nephrology centers in Germany employ a structured transition protocol, and if so, at what point it is initiated. We are documenting the structure of transition plans, including courses of action, the type of participant (such as physicians, psychologists, nurses, or case managers), and the financing of programs based on the study by Bethe and Haffner [14]. In centers without a structured transition protocol, we aim to explore how transitions are organized and why no transition program has yet been initiated. Firstly, we will compare how on-site staff evaluate structured versus unstructured transition of kidney-transplanted adolescents to adult medicine and will conduct surveys on the need for possible improvements. Secondly, by implementing a prospective, multicenter two-arm randomized intervention study, we plan to clarify whether a structured transition program with supplementary smartphone apps can improve the outcome of transitioning patients.

## Methods/Design

### Subjects

The study was approved by the research ethics committee of the Hannover Medical School (approval number: 6660). Informed consent will be obtained from the adolescent patients, as well as from their parents. The study cohort will include male and female patients who have undergone a kidney transplant who were between 16 and 22-years-old at study enrollment. There is a huge variation in age at transition in Germany; several centers make the transfer at 18 years whilst others do not transfer until the age of 22. For inclusion the patient must be: at least 14 months away from expected transfer to adult medical care, at least three months post-transplantation, capable of operating a smartphone, and able to provide informed consent. Exclusion criteria are: signs of acute transplant rejection or indications of a need for dialysis in the foreseeable future (since transplant survival is a study outcome variable), as well as mental incapacity (since it may prevent complete and independent use of the apps). Patients will be randomized into two groups: an intervention group that receives case management by the Berliner TransitionsProgramm, which is supported by a smartphone (Samsung Galaxy Y, Samsung, Seoul, South Korea) with two apps; and a control group transferred according to the established protocols of the center. There are centers in Germany which already practice some kind of transition management, however, no center actually uses smartphone apps or case management. Our interventions can also therefore improve outcome in centers that already practice active transition. Because of the center-based stratification used in this study, patients from German centers actually performing transition will be distributed equally amongst both groups.

### Sample size calculation

We first estimated the likely therapy adherence rate based on a small group of patients on immunosuppressive therapy. Data from Hannover Medical School showed that among six selected patients, three showed very good compliance, with a mean coefficient of variation (%) of 0.13 (±0.03), whereas poor compliance was observed in the three remaining patients, with an average coefficient of variation (%) of 0.5 (±0.03). These values were taken as roughly predictive of the variance we would observe in our study. We will receive additional data from phase I on which to base the sample size assumptions. Assuming a population of 50% with very good compliance, 25% with average compliance, and 25% with poor compliance, we simulated a mixed distribution with the statistical values above and calculated an average coefficient of variation (%) of 0.26, with a standard deviation of 0.15. Our expectation is that the intervention will increase the ratio of patients with good compliance. For that reason we assumed the following positively shifted distribution (%) for the intervention group: 70:30:0 of very good:average:poor compliance. This distribution corresponds to a coefficient of variation (%) of 0.181 with the same standard deviation of 0.15. Assuming a two-sided type I error of 5%, a Student’s two-sample t-test will have 80% power under the planning assumptions if n = 57 patients per group are randomized. Hence, a total of 114 patients must be recruited for this trial. We supposed a negligible dropout rate since it is critical that transplant patients receive ongoing care and attend their treatment centers regularly. In addition, we believe that providing free smartphones will increase adherence. In the unexpected case of too many dropouts, we will extend the recruitment phase to ensure sufficient patients are included in the trial throughout the complete follow-up period.

### Study design

**Figure 2:**
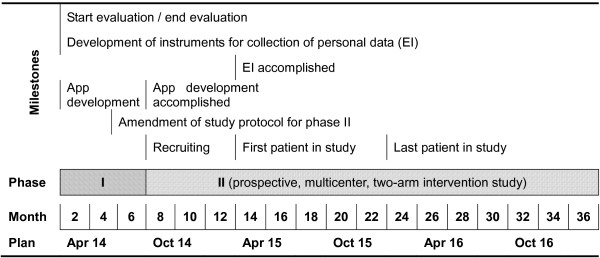
**Overview of study milestones, phases, and timeline.**

The study is a composite of two phases, as shown in Figure 2. In phase I, which is currently ongoing, we are conducting retrospective and prospective structured data collection and data analysis of the present medical care situation and care providers’ assessments of it. The progress of patients who have been transferred within the last three years and have been receiving medical treatment for at least a year are being collected in a retrospective anonymized manner and evaluated. Additionally, we are developing and piloting data collection tools (questionnaires, interview guidelines, and smartphone apps) for phase II of the study, which consists of an open, prospective, multicenter two-arm, randomized intervention analysis.

### Phase I

A retrospective analysis of the existing structures is carried out on-site at each of 17 pediatric nephrology centers, together with prospective questioning to assess the transition mechanisms. We follow a structured analysis of the existing transition procedures (initiation, beginning, financing, participants, documentation, and experiences) and assess healthcare providers’ views of their current institutional transition protocols based on qualitative (personal) expert surveys. Healthcare provider assessments include three interviews: one with a physician at each center, one with each center’s auxiliary nursing unit, and one with each center’s psychologist or social worker. The interviews are being analyzed in following recommendations made by Mayring [15] to define ways of recruiting subjects for the prospective study.

Information regarding each patient’s medical care and treatment course are registered via an anonymized questionnaire for patients who transferred in the years 2011 and 2012 from all of the participating pediatric nephrology centers, covering before and after the transfer. Treatment course variables include estimated glomerular filtration rate (eGFR), coefficient of variation (%) of immunosuppressive levels, and incidence of acute rejection and transplant loss. Additionally, an online transition questionnaire for physicians and nephrologists is being developed; it will be distributed by the German Society for Nephrology.

### Transfer supporting apps

We will employ apps designed to support the documentation and transfer of clinical data and to facilitate appointment scheduling. The apps are being developed for iOS and Android smartphones, with a diary function in cooperation, with the Children’s Aid Association for Organ Transplantation - Sportsmen for Organ Donation Registration Society (‘*Verein Kinderhilfe Organtransplantation - Sportler für Organspende e.V.*’ *(*KiO)). There is a pre-existing app that, in conjunction with KiO, is being extended for monitoring of clinical values (Kinderhilfe Organtransplantation, Frankfurt, Germany). In addition, a new app to organize appointments and communicate with the case manager will be developed to complement the Berliner TransitionsProgramm (Synectic, Berlin, Germany). The apps are currently being piloted with some patients.

### Phase II

Phase II of the study will be a real world, prospective, multicenter, randomized two-arm intervention, in which an intervention and a control group will be compared. In the control group, adolescents will go through transition as established in their center. In the intervention group, adolescents will be transitioned using structural elements from the Berliner TransitionsProgramm (case manager, transition interviews, and so forth) and with the smartphone apps developed during phase I.

An on-site physician involved in the study will obtain consent from patients that meet the inclusion and exclusion criteria, and also from parents for patients under 18 years of age. Patients will be enrolled at least 14 months before the study termination point and will be randomized into intervention and control groups. Identification of eligible patients began in the first year of the study. The physician continuing with each patient’s medical care will be identified and contact will be established with all receiving physicians for both study groups. Patient and physician addresses are also being registered so that, should there be a change in care providers, contact can be maintained. Patients and family members will be interviewed at two time points using standardized instruments for collecting personal data; these are being developed in phase I of the study. If necessary, available validated tools will be adapted.

### Time points

**Figure 3:**
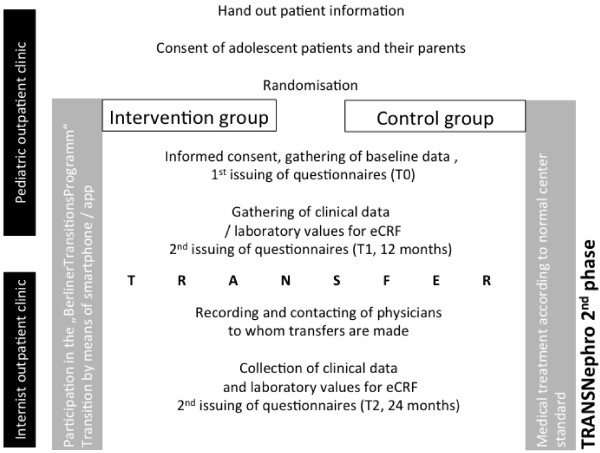
**Structure of the intervention study (Phase II).**

The phase II study timeline, summarized in Figure 3, begins with the start of transition and concomitant entry (E) into the study at time point T0 (baseline), and proceeds with a 12-month assessment at time point T1 and a 24-month assessment at time point T2. At T0 we will collect data for each group about each patient’s expectations for the transition, health status, time since transplantation, quality of life, satisfaction with medical care, therapy, adaptation to the disease, healthcare utilization, social environment, education, gender, age, and number of siblings. At T1, both groups will be evaluated for the transition planned, health status, quality of life, satisfaction with medical care, healthcare utilization, therapy system, and adherence. The intervention group will also be asked additional questions at T1 regarding their experience with the smartphone and app and how well they have adopted the program. After the T1 evaluation, each patient will be transferred to their respective adult care nephrologist. The following variables will be assessed at T2: evaluation of the transition, health status, quality of life, satisfaction with medical care, healthcare utilization, therapy system, education, employment, relationship status, and adherence.

Baseline data will be documented once, retrospectively. The course of immunosuppressive agent levels and eGFR, the number of acute rejection reactions (examined every four to eight weeks), and eventually the detection of donor-specific antibodies if evaluated by the nephrologist, will be recorded as surrogate parameters of therapy adherence. This diagnostic schedule is in accordance with standard German healthcare and would be followed regardless of participation in the study. No blood samples will be collected in addition to those collected in the normal course of care. Necessary data will be extracted retrospectively from patient files and added to the electronic clinical record file during the study.

### Protection against bias

Firstly, this is a prospective, randomized, intervention study. Secondly, patients are being assigned to groups by block randomization with a variable block length. Randomization will be performed for the most important prognostic factors, namely center, gender, and time after transplantation (less than five years or five years or more). The stratification for center will ensure that for both centers with and without experience in transition patients will be enrolled randomly to both groups. In this way, it will also ensure that the different approaches used in adult centers after transition will be distributed equally in both groups. Thirdly, the costs for the Berliner TransitionsProgramm are being absorbed for those patients whose health insurance company refuses to cover the costs. For all other patients, the costs will be regulated by health insurance.

### Study endpoints

The long-term goal of this work is to improve adherence through a controlled transition process. The primary outcome variable of the study is therapy adherence, as indicated by individual variability in immunosuppressive agent levels. Immunosuppressive agent levels will be recorded at all regular follow-up examinations (every four to eight weeks). A coefficient of variation (%) will be calculated for each patient based on these examination data. These coefficient values will serve as a surrogate parameter for patient adherence. The individual coefficient of variation (%) for immunosuppressive agent levels is the endpoint of choice for this field of research [15].

The secondary endpoints of the study are change in pre- and post-transfer eGFR (using the Schwartz 2009 formula [16], Modification of Diet in Renal Disease formula [17], and cystatin-C levels), serum creatinine levels, transplant survival, patient survival, acute rejection reactions (presence of donor-specific antibodies and chronic-humoral rejection), transplant loss, death, patient satisfaction, healthcare utilization, and patients’ self-reported quality of life and social integration.

### Statistical analysis

The coefficients of variation (%) for immunosuppressive agent levels will be analyzed by analysis of covariance, with adjustment for immunosuppressive agent level at T0 and all stratification variables considered in the randomization, such that the analysis of covariance can be used to determine group differences. [18] Least squares means will be calculated for each group and the difference between the group means will be reported with a two-sided 95% confidence interval (CI). If the upper limit of the CI for the difference between the adjusted means (transition minus standard) for the therapy effect is less than 0, then lower variability in immunosuppressive agent levels and thus better adherence to therapy due to the new transition model is therefore proved. Sensitivity analyses will be carried out using multivariate regression models with consideration of additional prognostic factors (modeling after stepwise variable selection). Secondary dichotomous endpoints (such as transplant loss) will be assessed with multivariate logistic regression models.

All patients included in the study (intention-to-treat population) will be considered in the primary and secondary analyses described above. For patients who suffer a transplant loss, the immunosuppressive agent levels measured before the loss will be used. Using a conservative approach, patients with fewer than three immunosuppressive agent level datum points will be coded with the largest coefficient of variation (%) in their respective therapy group. The study began recruiting patients as from 1 April 2014 (pre-screening).

## Discussion

This study will provide fundamental data about medical care structures in the transition of adolescent nephrology patients from pediatric to adult care. It is designed to test a new transition model using case management and modern telemedicine. According to the interventions used (case management and smartphone apps), our randomized controlled trial is an open unblinded study. This may influence the patient outcomes after 12 and 24 months. However, a blinded design is not possible in this kind of a trial.

To the best of our knowledge, there are no comparable randomized prospective studies in the literature. The major aim of the study is to utilize improved medical care structures, with the long-term goal of improving transplant survival and thereby reducing morbidity and mortality. Upon completion, this study ultimately aims to establish the Berliner TransitionsProgramm, combined with use of apps, as a medical care standard in the transition of young patients who have received a kidney transplant. Once the study has been completed, the apps will be made available throughout Germany. This work has the potential to optimize the medical care of adolescents during the critical pediatric-to-adult care transition phase.

## Trial status

Phase I of the study is in progress. Recruitment has started in November 2014 and is expected to finish in November 2015. The healthcare currently in place is being analyzed and the tools to be used in phase II are being reviewed.

## Abbreviations

CI: Confidence interval
eGFR: Estimated glomerular filtration rate
eCRF: Electronic Case Form
KiO: Kinderhilfe Organtransplantation
KfH: Kuratorium für Dialyse und Nierentransplantation.
